# Function of Vivid Coloration of Terrestrial Isopods from the Point of View of an Avian Predator

**DOI:** 10.3390/insects16070662

**Published:** 2025-06-25

**Authors:** Barbora Ďurajková, Petr Veselý, Ivan Hadrián Tuf

**Affiliations:** 1 Department of Ecology and Environmental Sciences, Faculty of Science, Palacký University Olomouc, 77900 Olomouc, Czech Republic; 2 Department of Zoology, Faculty of Science, University of South Bohemia, 37005 České Budějovice, Czech Republic; petr-vesely@seznam.cz

**Keywords:** antipredatory strategy, aposematism, warning signal

## Abstract

The ability of terrestrial isopods (Crustacea: Isopoda: Oniscidea) to protect themselves effectively from predation by birds has never been tested. They are equipped with glands producing chemical substances; moreover, some species show conspicuous coloration, which might suffice as an aposematic signal. We evaluated the palatability of isopods to birds. We tested the responses of *Parus major* captured in the wild (and thus possessing some experience with common native isopod species) to the following isopod species: *Porcellio scaber* (native, inconspicuous), *Oniscus asellus* (native, moderately conspicuous), *Armadillo officinalis* (non-native, moderately conspicuous), *Armadillidium versicolor* (native, conspicuous), and *Armadillidium gestroi* (non-native, conspicuous). We compared bird responses to isopods with reactions to the *Blaptica dubia*, an edible roach very similar to isopods in size and appearance. Isopods were better protected from bird attacks than roaches; however, their color pattern did not affect the level of protection. Birds were able to differentiate isopods from the roach; in experiments, where we presented isopod and roach individuals together, the birds hesitated longer in attacking and observed both prey items for a longer time. Non-native species either profited from the generalization of the protection of native isopods or from neophobia. Some isopods elicited significantly more discomfort behavior in birds, suggesting differences in the chemical protection among the tested species.

## Simple Summary

The ability of terrestrial isopods to protect themselves effectively from predation by birds has never been tested. Some species show conspicuous coloration, which might suffice as an aposematic signal. We tested the responses of Great Tits captured in the wild to five isopod species prey, we compared bird responses to isopods with reactions to the Guyana Spotted Roach, very similar to isopods in size and appearance. Isopods were generally better protected from bird attacks than roaches; however, their color pattern did not affect the level of protection. Birds were able to differentiate isopods from the roach, very likely using detailed features like antennae shape or manner of locomotion as a cue. In experiments, where we presented isopod and roach individuals together, the birds hesitated longer in attacking and observed both prey items for a longer time.

## 1. Introduction

Arthropods represent a group commonly armed with specialized chemical substances that serve both as a defense against predators and also in capturing prey, as can be seen in the case of the Hymenoptera’s sting apparatus or in the poison glands of centipedes [1]. Nevertheless, the use of chemicals for purely defensive purposes appears to be frequent across many arthropod species [1]. Apart from venom glands, chemical defense includes a broad spectrum of repugnatorial or odoriferous integumentary glands, the secretions of which are not injected, but applied topically in the form of liquid or as a vapor [2]. Such glands have been found in centipedes [3], millipedes, arachnids [1,4], and many species of insects [5] as well as crustaceans [6]. A detailed description of arthropod defensive secretion was summarized in Roth and Eisner [2]. Additionally, the bodies of some moth and butterfly larvae store secondary plant compounds to enable the manufacture of their own toxins [7]. Predators can detect toxins from their potential prey and avoid attack, or consequently spit out or regurgitate the unpalatable prey. Deterrent chemicals, thus, may protect arthropods from being swallowed when attacked [8].

Nevertheless, the prey usually minimizes the risk of being attacked or even swallowed and regurgitated and thus species that are unprofitable prey often display their unpalatability for predators through aposematic signals [9]. Respectively, natural selection, via predation, favors warning signals helping prey to inform a potential predator about its protection. The toxicity or unprofitability to a potential predator is mostly signaled through conspicuous coloration or patterning [10], but specific behavior or acoustic signals can be used [11]. This type of coloration is easy to learn [12], recognize and memorize by predators [13]. Aposematic color patterns consist of two types of contrast [14]. While luminance contrast describes dissimilarity between the light reflection of the object and its background, chromatic contrast indicates differences in hue or intensity of color between an object and the background on which it occurs [15,16]. Experiments with avian predators have indicated that color contrast is crucial to the effectiveness of aposematic signals. Nevertheless, the visual ability of predators differs according to their color discrimination capabilities [14], whereas bird and reptile sight is tetrachromatic [17]. The luminance contrast component of the aposematic coloration of prey can also function as an effective warning signal, even for color-blind predators [14].

Aposematic patterns provide advantages to both prey and predators, which may have facilitated their evolution from ancestrally cryptic coloration [18,19]. The selection of the warning coloration could also be affected by prey profitability. Highly profitable prey is often consumed by predators who prioritize nutritional benefits despite the negative effects of toxins [20,21]. Thus, the benefits of toxicity signaling through ostentatious coloration may be decreased in larger prey [22]; nevertheless, this relationship also depends on the size of the predator. Meta-analyses of different taxa have shown a positive relationship between the strength of chemical defenses (i.e., toxicity level) and the overall conspicuousness of the animal. This supports the suggestion that aposematism is a quantitatively honest signal [23]. Warning coloration is widely utilized by various insects [10,24], arachnids [4], as well as millipedes [25].

In the case of terrestrial isopods, there is little evidence of the use of chemical defense. Tegumental glands were described in detail by Gorvett [26,27,28,29]. Although numerous and varied glands can be found in almost every part of the isopod body, the defense function was suggested mostly for deeply lobed glands, which occur in the abdominal segments, uropods, and lateral plates. Their proteinaceous, stinking secretion is induced only by a violent stimulus (cf. types of violence in [29]). Gorvett [29] stated that the secretions of the lobed glands are primarily directed against spiders as the main predators of isopods. He proved that most spiders considered isopods as distasteful and avoided their consumption. The spider’s bite triggered the release of a secretion from the openings of the isopods’ glands. Thus, the localization of lobed glands ensures the maximum effect against the attacking spider. Spiders also rejected food material marked by the fluids of woodlice [29]. Those glands can also serve as a defense against scorpions [30], centipedes [31], ants [32], or shrews [33,34]. Herold [30] suggested that the weak development of the lobed glands in myrmecophilous genera such as *Platyarthrus* Brandt, 1833 and *Lucasius* Kinahan, 1859 is caused by their coexistence with ants, providing them protection. This supports Gorvett’s claim about the correlation between the degree of distastefulness and the level of development of such lobed glands [29].

Although aposematic coloration is widespread among many arthropods, there is little evidence for its function in terrestrial isopods. Schmalfuss and Ferrara [35] reported that a dark background color contrasting with whitish spots and stripes in several African isopod species has no intraspecific function and serves as a defence against visually orienting predators by optically dissolving the body silhouette. Levi [36] compared three unrelated species with bright red spots on a glistening black body (the pillbug *Armadillidium klugii* Brandt, 1833, the widow spider *Latrodectus tredecimguttatus* (Rossi, 1790), and the pill millipede *Glomeris pulchra* CL Koch, 1847), all living near Dubrovnik, Republic of Croatia. Levi suggests that these species use Müllerian mimicry, and their chemical defense and conspicuous coloration are directed against attacks from nocturnal mammals and reptiles (during cloudless Mediterranean summer nights), and avian predators such as thrushes and gallinaceous birds [37].

Birds possess among the most advanced color vision abilities in the animal kingdom [38]. Avian retinas are among the most complex of all vertebrates, allowing most birds to obtain information from the environment primarily via their visual sense [39,40]. Their tetrachromatic color vision is based on four visual pigments and has been described in detail [38,41,42,43,44,45]. Avian predators can learn how to avoid unpalatable prey based on their conspicuous coloration [13,20,46,47,48] or contrasting patterns [49,50]. Additionally, some studies have reported that aposematic prey remain relatively unharmed after bird attacks [51,52].

Moreover, birds have a remarkable ability to respond to chemicals found in their environment during activities such as foraging, navigation, or nest building (e.g., [53,54,55,56,57]). Avian species have also developed chemosenses such as olfaction [58,59], taste [60,61], and a trigeminal system [58,62], which all help them process chemical sensations. One function of those chemosenses is to detect and obtain suitable prey [63]. Birds can differentiate between palatable and unpalatable prey and use taste to control their chemical intake depending on the defense levels of the distasteful prey [64]. This corresponds with the finding that domestic chicks taste and reject aposematically colored prey [12,65].

The coloration of many isopods varies from grey to dark brown or even dark grey; thus, we can consider them cryptic-colored species. On the other hand, brightly colored species are also well known. Some isopods have bright yellow dots with unknown function on the dorsal part of the pereon. Our research aim was to examine whether the conspicuous coloration of terrestrial isopods could serve as a warning signal for a visually oriented predator, the Great Tit. In the present study, several species of isopods, with different colorations, and different probabilities of the Czech Great Tits having encountered them in the wild, were tested.

We tested the following hypotheses:

- All isopod species are protected against attack by birds better than edible roach.
- Isopod species with conspicuous coloration are better protected against attack by birds than inconspicuous species.
- Birds do not show any disgust after eating isopods.
- Birds prefer attacking roaches rather than isopods when encountering both simultaneously.

## 2. Materials and Methods

### 2.1. Prey

**Figure 1:**
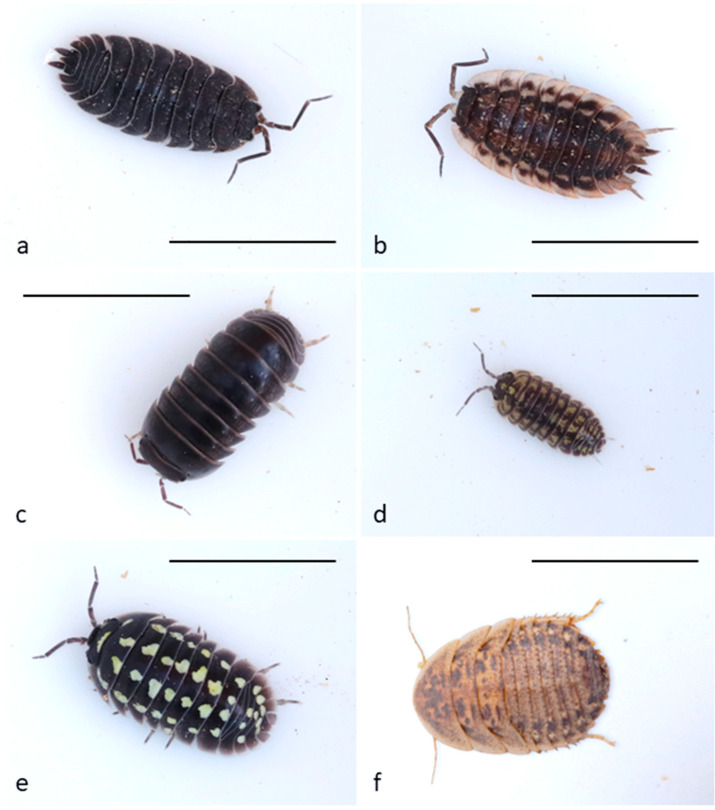
Tested prey types: (**a**) *Porcellio scaber* (scaber), (**b**) *Oniscus asellus* (asellus), (**c**) *Armadillo officinalis* (officinalis), (**d**) *Armadillidium versicolor* (versicolor), (**e**) *Armadillidium gestroi* (gestroi), (**f**) *Blaptica dubia* (roach); scale is 1 cm.

We used five species of terrestrial isopods (Figure 1a–e):

- The Common Rough Woodlouse *Porcellio scaber* Latreille, 1804 (coded as scaber throughout the text): Body size up to 17 mm, nearly twice as long as wide. The body is strongly irregularly tuberculate. Variable in color. Usually, dark slate grey, sometimes brightly mottled cream, brown, orange, or red forms are found [66]. Distributed across Central and Western Europe but introduced to most countries [67]. Common in the Czech Republic.
- The Common Shiny Woodlouse, *Oniscus asellus* Linnaeus, 1758 (coded as asellus throughout the text): Body size up to 18 mm, about twice as long as wide. Usually, shiny slate grey with irregular lighter patterns and two rows of yellow patches [66]. Distributed in Northern and Western Europe, east to Finland, Poland, and Ukraine. Also found in Madeira and the Azores. Introduced to America [68]. Common in the Czech Republic.
- *Armadillo officinalis* Duméril, 1816 (coded as officinalis throughout the text): Size of body up to 20 mm, slate grey in color [69]. Distributed in the Mediterranean and western Black Sea coasts [68]. Not occurring in the Czech Republic.
- *Armadillidium versicolor* Stein, 1859 (coded as versicolor throughout the text): Body size 10 mm long. Dark coloring varies from black-brown to olive-brown, with light spots forming 5 regular rows on the dorsal side and pale-yellow margins of epimeres [70]. Found in Eastern Europe [68]. Rare in the Czech Republic, but locally abundant.
- *Armadillidium gestroi* Tua, 1900 (coded as gestroi throughout the text): Body size up to 20 mm. Color dark or light brown with vivid yellow spots forming 4 regular rows. Found in northwest Italy [68]. Not occurring in the Czech Republic.

*Porcellio scaber*, *O. asellus*, and *A. versicolor* were hand-picked in an urban area of the city of Olomouc, Czech Republic. *Armadillidium officinalis* and *A. gestroi* were obtained from a local breeder. Isopods were kept under natural light in laboratory conditions (ca. 19–22 °C) in 17 × 17 × 8 cm plastic boxes with a thin layer of plaster and leaf litter to maintain humidity and fed on carrot ad libitum.

As a control baseline prey, we used the Guyana Spotted Roach (*Blaptica dubia* (Serville, 1839); coded as roach throughout the text, Figure 1f). This species originates from Argentina, it is therefore novel for the European wild caught Great Tits. We used the third larval instar, which is very similar in appearance to isopods, brown-grey with light and dark spots, and a length of 10–18 mm. The Guyana Spotted Roach may use the smell of volatiles derived from their food as a defensive secretion. The experimental individuals were fed carrots. Despite nothing being known about their ability to produce defensive chemicals, or how they spread through the air, this species is palatable to, and commonly eaten by, Great Tits in laboratory experiments [71,72,73].

### 2.2. Predator

We used the Great Tit (*Parus major* Linnaeus, 1758) as a predator as it is a common insectivorous passerine with a broad array of invertebrate taxa in its diet [74]. It is commonly used in behavioral experiments testing the efficacy of the antipredator signals of invertebrates [72,74,75,76] owing to its ability to quickly accustom itself to laboratory conditions. The birds used for the experiments were adults captured in the wild at a winter-feeding spot within the environs of the city of České Budějovice (Czech Republic). Captures were conducted from October to November 2022. Each bird was tagged with an aluminium ring with a unique code and transported in captivity. Bird was placed in a birdcage with access to freshwater and food (sunflower seeds and mealworms *Tenebrio molitor* Linnaeus, 1758). Birdcages were deposited in a room with maintained natural daylight and temperature similar to outside conditions. Altogether, we used 80 individual birds. Each bird was kept in the cage one day prior to the experiment to get habituated to captivity. Birds were caged individually to avoid any social stress. Each bird was tested only once and released back into the wild at the end of the experiment.

### 2.3. Experiment

Two hours before the start of the experiment, each individual was taken to the experimental cage (70 × 70 × 70 cm), which consisted of a wooden frame, wire mesh, and a front wall made of one-way glass. Inside the cage was a perch, a bowl of water, and a circular feeding tray carrying six cups (5.5 cm diameter) with a white bottom on which prey was presented. The distance between the perch and the feeding tray was 40 cm. The birds were left with only clean water and five mealworms, as had been performed in previous studies [76,77], to encourage an increased interest in experimental prey but at the same time avoid inducing stress from starvation. The bird spent two hours before the experiment in the experimental cage to get accustomed to the cage and to associate the movements of the rotating tray with the provisioning of food. The birds usually looked at the offered prey immediately when the tray rotated. The experimental cage was placed in a dark room, lit by a single lamp (15 W) simulating daylight, including UVA rays. We conducted two types of experiments. In the single-prey treatment, each prey was offered in one (middle) cup of a circular feeding tray. The moment the Great Tit started looking for food in the cups was considered the beginning of the experiment. The bird was first given a mealworm, which was used to control interest in foraging. When the bird had completely swallowed the mealworm, one isopod individual was provided. With every bird, a series of 5 trials (in order: mealworm, isopod, mealworm, isopod, etc.) was performed to avoid the effect of neophobia [78]. Each trial with one isopod lasted five minutes, and the experiment continued only after mealworm consumption.

The choice tests were conducted similarly, the only difference being that the bird was presented with two types of prey at the same time. Roaches were used as second prey. Mealworms served only as an appetite indicator. Thus, a scheme of mealworm—cockroach + isopod—mealworm—cockroach + isopod (etc.) was followed. In this type of experiment, we used the prey types *Blaptica dubia* (coded as roach (+scaber) throughout the text), *Porcellio scaber* (coded as scaber (+roach) throughout the text), *Blaptica dubia* (coded as roach (+gestroi) throughout the text) and *Armadillidium gestroi* (coded as gestroi (+roach) throughout the text). We chose only these two species of terrestrial isopods because of their different conspicuousness of coloration and because both were sufficiently abundant for experimentation.

### 2.4. Data Analyses

We recorded the bird behavior with the help of Behavioral Observation Research Interactive Software (BORIS, ver. 9.4.1). We scored multiple bird behaviors, four of which were used in further analyses, as they related to the prey. (1) attacking the prey—any manipulation with the prey, carrying the prey in the bill, (2) eating the prey—consumption of at least part of the prey body, (3) observing the prey—looking at the prey from distance, (4) discomfort behavior—cleaning bill, drinking water, flushing feathers.

Analyses were performed in R ver. 4.1.2. Firstly, we ran a generalised linear mixed effect model (GLMM, command glmer in R package lme4 [79]) to test the effect of the prey type (values: scaber, scaber (+roach), asellus, officinalis, versicolor, gestroi, gestroi (+roach), roach, roach (+scaber), roach (+gestroi)) on the occurrence of attack on the particular prey (coded binomially). As each bird experienced five consecutive trials, the bird′s identity was included in the model as a random factor. We used the Likelihood ratio test for binomial data to compare the models in forward stepwise selection (Chi-square test). Further, we used the Fisher LSD post hoc test with Tukey correction for repeated comparisons to compare particular prey types.

We further analyzed the edibility of particular prey types by analyzing the occurrence of eating after attacking the prey. We ran a generalized linear mixed effect model (GLMM, command glmer in R package lme4) to test the effect of the prey type on the occurrence of eating (binomially coded, bird ID included as a random factor). We used the Likelihood Ratio Test for binomial data to compare the models in forward stepwise selection (Chi-square test). Further, we used the Fisher LSD post hoc test with Tukey correction for repeated comparisons of offered prey.

Thirdly, we analyzed the time spent observing the prey from a distance to show the effect of familiarity or decision-making. In this case, we were not able to distinguish which prey the bird looked at in the choice tests. Therefore, prey scaber (+roach) and roach (+scaber) as well as gestroi (+roach) and roach (+gestroi) were coded as one prey type. As these data followed the Gaussian distribution of errors, we ran a mixed-effect linear model (LMM, command lmer in R package lme4) to test the effect of the prey type on the time spent observing the prey. We used the Likelihood ratio test for normal data to compare the models in forward stepwise selection (Chi-square test). Further, we used the Tukey HSD post hoc test with Tukey correction for repeated comparisons to compare particular prey types.

Lastly, we analyzed how often the bird showed any form of discomfort (cleaning bill, drinking water, flushing feathers) after an attack on particular prey. We ran a generalized linear mixed effect model (GLMM, command glmer in R package lme4) to test the effect of the interaction of prey type and whether the particular prey was attacked or not on the number of signs of discomfort (Poisson distribution of data). We used the Likelihood Ratio Test for Poisson data to compare the models in forward stepwise selection (Chi-square test). Further, we used a post hoc test for the Poisson distribution of data with Tukey correction for repeated comparisons to compare the offered prey.

## 3. Results

### 3.1. Attacking of Prey

**Figure 2:**
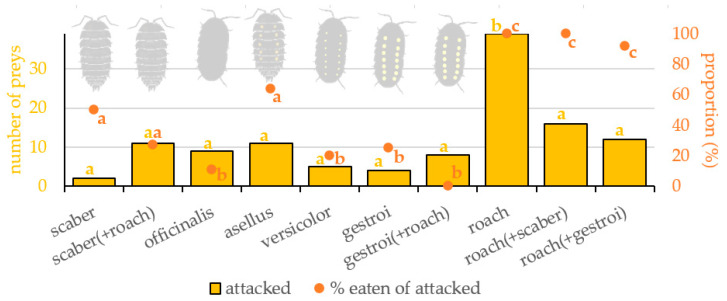
Number of preys of particular type attacked (yellow) and proportion of them eaten (orange) by Great Tits (*Parus major*). Tested prey types: scaber—*Porcellio scaber*, asellus—*Oniscus asellus*, officinalis—*Armadillo officinalis*, versicolor—*Armadillidium versicolor*, gestroi—*Armadillidium gestroi*, roach—*Blaptica dubia*. Combination of two prey refers to preference experiments when two prey items were presented simultaneously. Yellow and orange letters indicate significant differences.

The prey type significantly affected the occurrence of attacks on it (Table 1 and Figure 2). Roach was attacked significantly more often than scaber (z = 4.345, *p* < 0.001), asellus (z = 3.213, *p* = 0.040), officinalis (z = 3.408, *p* = 0.021), versicolor (z = 3.924, *p* = 0.003), and gestroi (z = 4.013, *p* = 0.002). There was no difference in the attack occurrence among all isopod forms (z < 1.670, *p* ˃ 0.798).

Roach presented together with scaber was attacked equally often as scaber presented together with roach (z = 1.779, *p* = 0.731). Roach presented together with gestroi was attacked equally often as gestroi presented with roach (z = 1.542, *p* = 0.864). Scaber was attacked equally often when presented alone or when presented with a roach (z = 1.531, *p* = 0.869). Gestroi was attacked equally often when presented alone or when presented with a roach (z = 0.434, *p* = 0.999). Roach was attacked significantly less often when presented with scaber than when presented alone (z = 3.498, *p* = 0.016). Roach was attacked a little less often when presented with gestroi than when presented alone (z = 3.018, *p* = 0.071).

### 3.2. Eating of Prey

The prey type significantly affected consumption of the already attacked prey (Table 1 and Figure 2). Roach was eaten significantly more often than scaber (z = 3.001, *p* = 0.016), asellus (z = 2.659, *p* = 0.042), officinalis (z = 5.012, *p* < 0.001), versicolor (z = 5.123, *p* < 0.001), and gestroi (z = 3.568, *p* = 0.002). Scaber was eaten more often than officinalis (z = 2.997, *p* = 0.026), versicolor (z = 3.203, *p* = 0.009), and gestroi (z = 2.204, *p* = 0.049), and equally often as asellus (z = 0.982, *p* = 0.998). Asellus was eaten more often than officinalis (z = 3.259, *p* = 0.004), versicolor (z = 3.151, *p* = 0.008), and gestroi (z = 2.653, *p* = 0.039). There was no difference among the eating of officinalis, versicolor, and gestroi (z < 1.090, *p* ˃ 0.998).

Roach presented together with scaber was eaten more often than scaber presented together with roach (z = 4.100, *p* = 0.004). Roach presented together with gestroi was eaten more often than gestroi presented with roach (z = 4.993, *p* < 0.001). Scaber was eaten a little more often when presented alone than when presented with a roach (z = 1.872, *p* = 0.062). Gestroi was eaten equally often when presented alone or when presented with a roach (z = 1.309, *p* = 0.956). Roach was eaten equally often when presented with scaber, when presented with gestroi, or when presented alone (z < 0.523, *p* ˃ 0.9).

### 3.3. Observation of Prey

**Figure 3:**
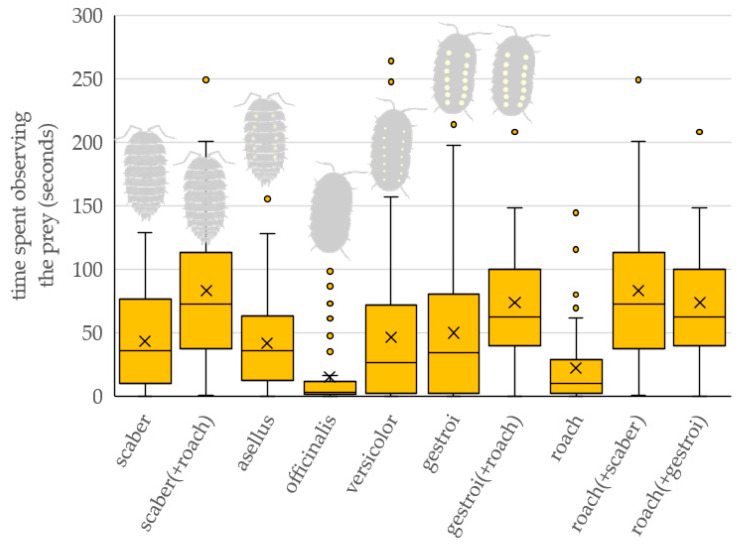
The total time the bird spent observing the prey, or prey combination, from a distance. Tested prey types: scaber—*Porcellio scaber*, asellus—*Oniscus asellus*, officinalis—*Armadillo officinalis*, versicolor—*Armadillidium versicolor*, gestroi—*Armadillidium gestroi*, roach—*Blaptica dubia*. Combination of two prey refers to preference experiments when two prey items were presented simultaneously.

The prey type significantly affected the total time the bird spent observing the prey or prey combination (Table 1 and Figure 3). Birds spent significantly longer observing the roaches presented in combination with scaber and gestroi than they did officinalis (roach (+scaber): z = 4.538, *p* < 0.001; roach (+gestroi): z = 3.902, *p* = 0.004) and roach (roach (+scaber): z = 4.103, *p* = 0.002; roach (+gestroi): z = 3.466, *p* = 0.017).

### 3.4. Signs of Discomfort

**Figure 4:**
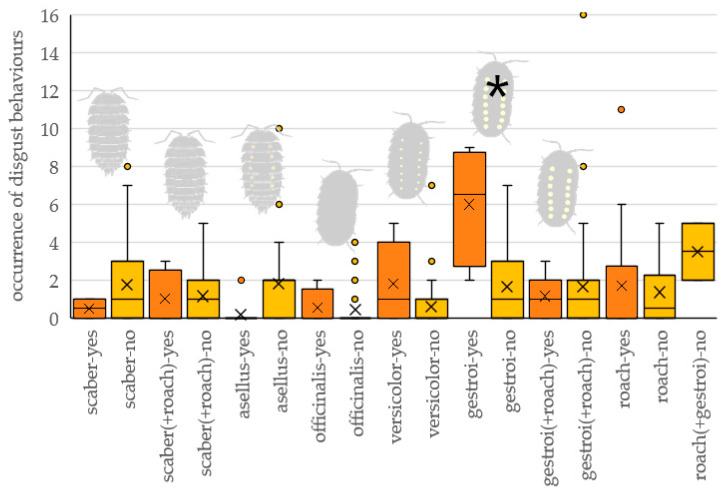
The number of signs of discomfort performed by tested birds attacking (yes, orange) and nonattacking (no, yellow) particular prey types. Tested prey types: scaber—*Porcellio scaber*, asellus—*Oniscus asellus*, officinalis—*Armadillo officinalis*, versicolor—*Armadillidium versicolor*, gestroi—*Armadillidium gestroi*, roach—*Blaptica dubia*. An asterisk marks a significant difference in the number of discomfort signs between birds attacking and nonattacking prey. Combination of two prey refers to preference experiments when two prey items were presented simultaneously.

The interaction of prey type and the occurrence of attacking the prey significantly affected the number of signs of discomfort (cleaning beak, feather flushing, drinking) the tested bird performed (Table 1 and Figure 4). Birds showed discomfort most often when attacking the gestroi prey type, significantly more often than when they did not attack it (z = 5.508, *p* < 0.001); or significantly more often than when they attacked other prey types (scaber: z = 4.435, *p* < 0.001; asellus: z = 4.751, *p* < 0.001; officinalis: z = 4.840, *p* < 0.001; versicolor: z = 3.080, *p* < 0.001; roach: z = 5.247, *p* < 0.001). There was no difference in the number of signs of discomfort shown by birds when attacking and non-attacking other prey types (scaber: z = 1.257, *p* = 0.999; asellus: z = 3.193, *p* = 0.111; officinalis: z = 0.575, *p* = 0.999; versicolor: z = 2.854, *p* = 0.264; roach: z = 0.845, *p* = 0.999).

## 4. Discussion

### 4.1. Attacking and Eating Prey

We found out that the type of prey significantly affected the occurrence of bird attacks. The edible roach was attacked more often than the isopods. However, we did not find a statistical difference in attack occurrence among the five isopod forms. This is inconsistent with our hypotheses that no isopod species is better protected against attack and eating by birds than an edible roach. We also did not prove that isopod species with conspicuous coloration are better protected against bird attack than inconspicuous species.

The reaction to inconspicuous species of isopods differed from the reaction to the edible roach, which at first glance seems similar, differing mainly in the shape of the antennae. The use of such detailed characters in prey recognition has been demonstrated by Karlíková et al. [71]. In their study, it was tested whether Great Tits can discriminate between the edible roach (*B. dubia*) and the inedible firebug (*Pyrrhocoris apterus* (Linnaeus, 1758)) when their coloration was manipulated to be identical using paper sticker placed on their back. The study showed that some of the birds were able to recognize edible roaches and attacked only them [71]. It seems to be plausible to presume that the birds used other morphological cues related to differences between roaches and true bugs (differences in the shape of antennae or legs, body posture, etc.).

The birds tested in our study originated from the wild and probably had previous experience with common native species of woodlice (mainly *P. scaber* and *O. asellus*). Alatalo and Mappes [80] demonstrated that Great Tits initially attack all prey, but through aversion learning begin to avoid distasteful prey. Such aversive learning is particularly rapid for prey with higher levels of chemical defenses [81], which these species probably have [82,83]. The conspicuous visual appearance of *O. asellus* coloration may have increased the rate of memorization of unsuitable prey [84] and could be generalized as a color signal and used also for the protection of non-native *A. gestroi* and *A. versicolor* [85,86].

When presented alone or when presented with a roach, the isopods were attacked equally often. However, when a roach was presented together with isopods, it gained some level of protection. Birds probably can not distinguish between them and do not attack either of them. This is also in contrast with our hypothesis that birds prefer attacking roaches rather than isopods when encountering both simultaneously. On the other hand, the same results were found by Karlíková et al. [71]. The study showed that generally, Great Tits were not able to distinguish between inedible firebugs and edible roaches when their colorations were identical and they were presented together. The majority of birds attacked both prey, or attacked neither of them [71]. It seems that such a distinction between similar prey types is cognitively very demanding, and the bird cannot solve this task fast enough. This is suggested by the longer observation times of prey in our choice experiments.

Roach was eaten more often than all isopod forms, both presented alone or together with isopods. We found out that *P. scaber* and *O. asellus* were eaten more often than other isopod species. Still, all isopods are protected better than roaches, both from being attacked and being eaten. Both of these species are native to the experimental site, and it is likely that the tested birds meet with them, might have tasted them, and learned that woodlice are not very tasty. However, the optimization of decision-making strategies is influenced by a number of external factors, including current energy requirements [87]. In our laboratory arrangement, the bird may be motivated to forage on disadvantageous prey, as long as it is edible, as, unlike in the field, there is no alternative. We conducted the experiments during the autumn period, when Great Tits prepare for winter and some individuals engage in dispersal [88]. The foraging motivation of birds is generally higher before and during migration, which may also increase their willingness to attack even an unprofitable prey.

Neophobia may also play a part in the avoidance of at least some isopod forms. As we presented non-native species, at least some Great Tit individuals may have shown caution, and decided not to attack them. Neophobia can be overcome quite quickly, especially by individuals more prone to risk-taking over time [89]. Obviously, five repeated encounters did not suffice to overcome the neophobia in our case.

It cannot be ruled out that the birds distinguished their prey by scent. Their sense of smell is probably powerful enough [58], but woodlice usually emit odors only after mechanical or other irritation [83]. This possibility is therefore not very likely.

### 4.2. Observing Prey

When the roach was presented together with the isopod, birds spent a long time observing them from a distance. Birds spent more time observing the combination of roach and *P. scaber* and roach and *A. gestroi* than other types of prey combinations. Levi [36] states that isopods use chemical defense and conspicuous coloration directly against attacks from visual predators. Apparently, the appearance of all isopod species, even the cryptic ones, evokes in birds the memory of unpleasant experiences of meeting an isopod in the wild. Birds with such experience are much more cautious and spend more time choosing their prey. This is also related to the cognitive difficulty of recognizing all the characteristics. In addition to the shape of the antennae mentioned above, the woodlice and cockroaches also differ in the nature of their movements. It is therefore advisable to wait until the prey starts to move, which can take on the order of minutes for isopods [90].

### 4.3. Discomfort Behavior 

When birds attacked *A. gestroi,* they showed an increased number of discomfort signs in comparison to other prey types. This is in contrast with our hypothesis that birds do not show any disgust after attacking and eating isopods. However, we did not find any difference in the number of disgust signs shown by birds when attacking and non-attacking other types of prey. It is obvious that isopods are protected from avian predators. But the reaction after the attack is not strong. Except with *A. gestroi*, Great Tits do not show disgusted behavior like rubbing their beaks or drinking water after eating isopods, which is a common reaction to chemically protected prey. As was mentioned above, isopods’ repugnatorial glands are primarily directed against invertebrates [29] or small insectivorous mammals [33,34]. It can therefore be assumed that the chemical protection is not significant regarding birds, but their aversion to isopods still occurs. We know nothing about the intensity of chemical defenses in terrestrial isopods, and we have very little information on the presence of defense glands in different woodlouse species. However, if we use the number of defensive glands in each species as a proxy for estimating their effectiveness, recent research [82] suggests that conglobating species (e.g., the family Armadillidiidae) are less chemically protected than species of the family Porcellionidae or Oniscidae, which is in contrast to our finding that *A. gestroi* elicits most discomfort after the attack. Nevertheless, even in the genus *Armadillidium*, there are differences in the smell intensity of defensive secretions [83].

## Figures and Tables

**Table 1:** Effects of tested predictors on particular response behaviors of birds (always mixed effect models with bird ID included as a random factor). DF refers to degrees of freedom, and the asterisk refers to the interaction of factors.

Response	Predictor	Data Type	Data Included	Chi	DF	*p*
attacking	prey type	binomial	all	37.575	9	<0.001
eating	prey type	binomial	only attacking	62.303	9	<0.001
observing	prey type	gaussian	all	31.97	7	<0.001
disgust	prey type * attack	poisson	all	12.256	9	<0.001

## Data Availability

The raw data supporting the conclusions of this article will be made available by the authors on request.
